# Evaluation of daily online contour adaptation by radiation therapists for prostate cancer treatment on an MRI-guided linear accelerator

**DOI:** 10.1016/j.ctro.2021.01.002

**Published:** 2021-01-14

**Authors:** Thomas Willigenburg, Daan M. de Muinck Keizer, Max Peters, An Claes, Jan J.W. Lagendijk, Hans C.J. de Boer, Jochem R.N. van der Voort van Zyp

**Affiliations:** Department of Radiation Oncology, University Medical Center Utrecht, The Netherlands

**Keywords:** Prostate cancer, MR-Linac, MRI-guided radiotherapy, Online contour adaptation, Adapt-to-shape, Radiation therapists

## Abstract

**Background and purpose:**

Magnetic resonance (MR)-guided linear accelerator (MR-Linac) systems have changed radiotherapy workflows. The addition of daily online contour adaptation allows for higher precision treatment, but also increases the workload of those involved. We train radiation therapists (RTTs) to perform daily online contour adaptation for MR-Linac treatment of prostate cancer (PCa) patients. The purpose of this study was to evaluate these prostate contours by performing an interfraction and interobserver analysis.

**Materials and methods:**

Clinical target volume (CTV) contours generated online by RTTs from 30 low-intermediate risk PCa patients, treated with 5x7.25 Gy, were used. Two physicians (Observers) judged the RTTs contours and performed adaptations when necessary. Interfraction relative volume differences between the first and the subsequent fractions were calculated for the RTTs, Observer 1, and Observer 2. Additionally, interobserver dice’s similarity coefficient (DSC) for fraction 2–5 was calculated with the RTTs- and physician-adapted contours. Clinical acceptability of the RTTs contours was judged by a third observer.

**Results:**

Mean (SD) online contour adaptation time was 12.6 (±3.8) minutes and overall median (interquartile range [IQR]) relative volume difference was 9.3% (4.4–13.0). Adaptations by the observers were mostly performed at the apex and base of the prostate. Median (IQR) interobserver DSC between RTTs and Observer 1, RTTs and Observer 2, and Observer 1 and 2 was 0.99 (0.98–1.00), 1.00 (0.98–1.00), and 1.00 (0.99–1.00), respectively. Contours were acceptable for clinical use in 113 (94.2%) fractions. Dose-volume histogram (DVH) analysis showed significant CTV underdosage for one of the seven identified outliers.

**Conclusion:**

Daily online contour adaptation by RTTs is clinically feasible for MR-Linac treatment of PCa.

## Highlights

• Adapt-to-Shape (ATS) workflows enable high-precision MR-guided radiotherapy.
• Online, manual contour adaptation is a time-consuming step in the ATS workflow.
• Trained radiation therapists adapt contours for prostate cancer treatment.
• CTV contours by radiation therapists are well-suited for MR-Linac prostate cancer treatment.

## 1 Introduction

The clinical introduction of magnetic resonance (MR)-guided linear accelerator (MR-Linac) systems has led to major changes in the workflows for treatment of various types of cancer [1], [2]. MR-Linac systems integrate an MR imaging scanner with a linear accelerator, enabling ‘online’ – when the patient is on the treatment table – imaging, contour adaptation, and treatment (re)planning [3]. To make optimal use of the capabilities of these systems, online treatment steps are added to the workflow. One example is deformable image registration (DIR) of pre-treatment and daily MR images. After registration, the pre-treatment contours are propagated to the daily MR scan, followed by manual adaptation to make them perfectly fit the anatomy of the day. This so called ‘Adapt-to-Shape’ (ATS) workflow is aimed at delivering the highest precision treatment to the patient, thereby potentially reducing toxicity and improving oncological outcomes [4], [5].

Consequently, these new online tasks increase the workload of those involved in the radiotherapy treatment of patients. With increasing numbers of patients being treated on MR-Linac systems worldwide, new approaches for the online workflow are being implemented. This also encompasses the transition of tasks from radiation oncologists to radiation therapists (RTTs). One of the most time-consuming steps in the ATS workflow for MR-Linac treatment is the manual adaptation of the propagated contours on the daily online MR scan by radiation oncologists. Since October 2019, RTTs in our centre are trained and certified to perform the online contour adaptation and to approve the contours for prostate cancer (PCa) treatment. Besides this, they also perform all other steps of the workflow, such as image registration, treatment planning, and approval of the treatment plan.

With the delegation of the contour adaptation task from radiation oncologists to RTTs, both need to be confident with the associated responsibilities. The purpose of this study was to evaluate the clinical prostate contours as adapted and approved by RTTs in the online MR-Linac setting.

## 2 Materials and methods

### 2.1 Patient characteristics

For this study 30 consecutive patients, who were treated for low or intermediate risk PCa (NCCN criteria) at the Radiotherapy Department of the University Medical Center Utrecht between January and March 2020, were included. All patients were part of an institutional review board approved registration and imaging study. Patients were treated with 5 fractions of 7.25 Gy over the course of 2.5 weeks on a 1.5 T Unity MR-Linac (Elekta AB, Stockholm, Sweden).

### 2.2 Offline workflow

Patients underwent a computed tomography (CT) and/or MR simulation scan prior to the first fraction. From January 30th, 2020 onwards, an MR-only workflow was implemented, discarding the need for a CT simulation scan (17 out of the 30 patients). Pre-treatment delineations were performed using the in-house developed software Volumetool® [6] by experienced radiation oncologists on the pre-treatment MR scan. The gross tumour volume (GTV) contained the MR visible tumour with a 4 mm isotropic margin for microscopic extension (GTV + 4 mm), excluding the organs-at-risk (OAR) [7]. The clinical target volume (CTV) encompassed the prostate body including the GTV + 4 mm. For intermediate risk patients, up to 1 cm of the seminal vesicles were included based on judgement of the treating physician. The planning target volume (PTV) included the CTV with a 5 mm isotropic margin. Intensity modulated radiotherapy (IMRT) treatment plans were created using the Elekta Monaco treatment planning system (Version 50.40.01, Elekta Inc., Stockholm, Sweden), prescribing a dose of 36.25 Gy to the PTV.

### 2.3 Online workflow

**Fig. 1:**
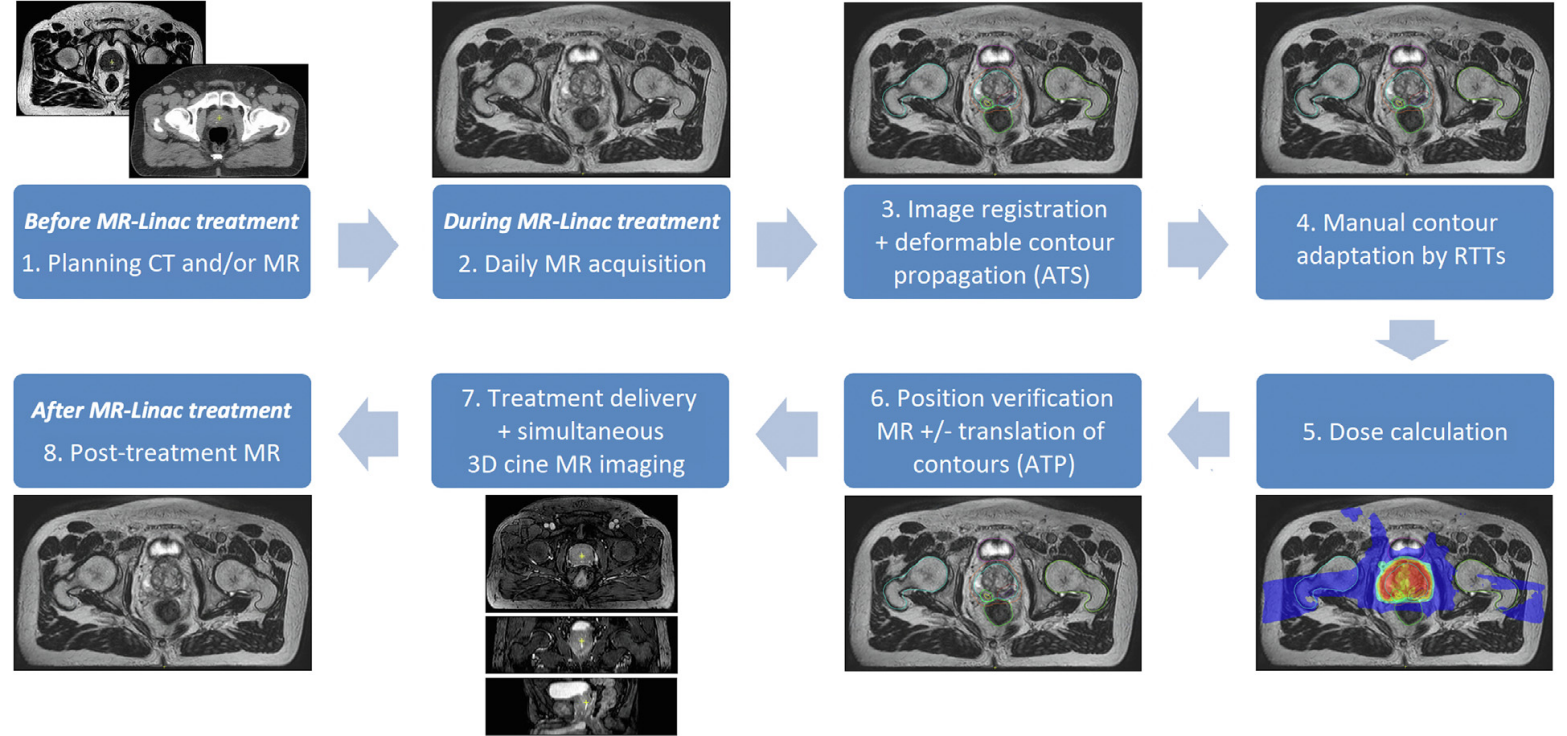
MR-Linac workflow for prostate cancer treatment. CT = Computed Tomography. MR = Magnetic Resonance. ATS = Adapt to shape. RTTs = Radiation therapists. ATP = Adapt to position.

The ATS workflow is depicted in Fig. 1 [4]. During each fraction, after positioning the patient on the treatment couch, a daily online T2-weighted MR scan was obtained with an acquisition time of 2 min, a field-of-view of 0.448x0.448x0.300 m, and a reconstructed voxel spacing of 0.8x0.8x2.0 mm^3^. For the CT-based workflow, during the first fraction the pre-treatment CT was registered to the online MR scan and the contours were propagated from the CT scan to the MR scan using DIR that is part of the Monaco treatment planning software for the Unity MR-Linac (Version 50.40.01, Elekta Inc., Stockholm, Sweden). For fractions 2 to 5, the online MR scan of the first fraction was registered to the daily MR scan and likewise the contours from fraction 1 were propagated to the daily MR. For the MR only workflow, contours were propagated from the pre-treatment MR to the daily online MR scan for all fractions. After DIR and contour propagation, the contours were checked and manually adapted by certified RTTs. During each first fraction, a radiation oncologist was present for approval of the adapted contours. For the remaining fractions, only in case of specific questions or concerns a radiation oncologist was present. After approval of the daily contours, the treatment plan was recalculated, and a position verification (PV) MR scan was obtained. In case the CTV was no longer covered by the PTV, a dose shift was applied, also known as ‘Adapt-to-Position’ (ATP) [4]. During dose delivery, 3D cine MR images were acquired for analysis of intrafraction prostate motion [8].

### 2.4 Training and certification of RTTs

To become a certified MR-Linac RTT, RTTs with either experience in treatment planning or clinical image processing, and preferably MRI experience, are trained both offline and online for 4–6 weeks. Training consists of general workflow training, image registration, contour adaptation, treatment planning, dose check, and treatment plan approval, depending on the profile of the RTT. The training for contour adaptation by RTTs consisted of two phases. During the first phase, RTTs performed 5 offline prostate CTV delineations on T2-weighted MR images. These were examined by an experienced PCa radiation oncologist. To improve their contouring skills, any disagreement on the contours was discussed between the RTT and the physician. In case the physician was satisfied with the offline contours, 15 online contour adaptations were performed in conjunction with a radiation oncologist. Once the online contour adaptations were performed satisfactorily, RTTs obtained their certificate and were allowed to perform the adaptations without direct supervision, with exception of each patient’s first fraction. In this patient cohort, contour adaptations were performed by a group of eight RTTs.

### 2.5 Contour adaptation timings

During each fraction, all steps of the online workflow were timed. The time taken for the contour adaptation by the RTTs was measured as the time interval between the start of manual adaptation and the start of treatment plan calculation. During this time, both the CTV as well as OAR contours within a 2 cm ring around the CTV contour were adapted.

### 2.6 Evaluation of contours

All 150 CTV contours were independently judged by 2 radiation oncologists (Observer 1 and 2) and adapted if deemed necessary. Both observers were blinded for each other’s adapted contours. Fraction 1 was chosen as a reference for interfraction analysis, since for each patient a radiation oncologist was present for approval of the contours during the first fraction and due to the fact that no ground truth was available for each fraction. Evaluation of the contours consisted of three parts. Firstly, CTV volumes were obtained using Volumetool® [6] and relative volume differences between the contours from fraction one and the contours from fraction two to five were calculated, separately for the RTTs and for Observer 1 and 2. Secondly, interobserver DSC per fraction was calculated between the RTTs and the observers as well as between both observers for fraction two to five, using MATLAB (version R2019a). Finally, a third ‘senior’ observer judged the RTTs contours on clinical acceptability, taking into account the extent of the adaptations (if any) that were performed by Observer 1 and 2 and expected interobserver variability in the base and apex region [9], [10]. All analyses were performed using R statistical software (version 3.6.2).

### 2.7 Dosimetric analysis

To estimate the effect on the CTV dose for outliers that needed more extensive, potentially clinically relevant adaptations as judged by Observer 3, dose-volume-histogram (DVHs) were calculated for the RTTs- and Observer-adapted contours, using the corresponding (RTTs contour-based) online dose distribution. Also, CTV D99% (dose to 99% of the volume) was calculated. These analyses were performed using Volumetool® [6].

## 3 Results

**Fig. 2:**
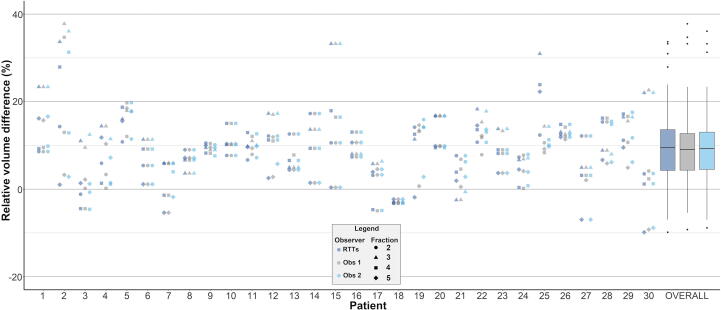
Intra-observer relative volume differences. Plots of relative volume differences for fraction two to five compared to fraction one for each patient separately and for all patients and fractions combined (“OVERALL”) for the RTTs (dark blue), Observer 1 (grey), and Observer 2 (light blue). For “OVERALL”, the boxplot boxes indicate the median (black horizontal bar), upper and lower quartile (vertical borders of boxes), and interquartile range (IQR). Black tails (error bars) indicate the lowest and highest value that is within the minimum and maximum value (minimum = lower quartile - 1.5*IQR and maximum = upper quartile + 1.5*IQR). Outliers are presented as black dots. RTTs = Radiation therapists. Obs = Observer.

**Fig. 3:**
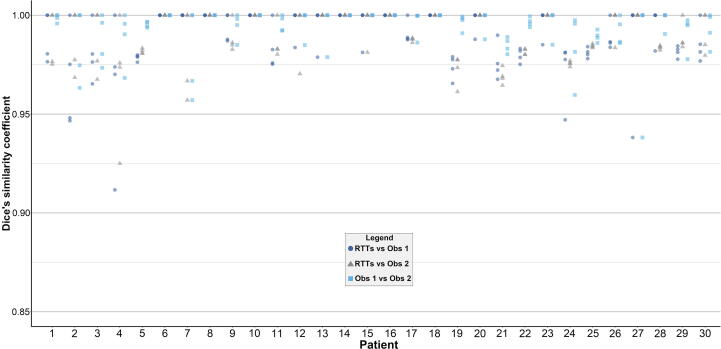
Interobserver dice’s similarity coefficients. Plots of interobserver dice’s similarity coefficients per fraction and patient (fraction two to five compared to fraction one), separately for RTTs versus Observer 1 (dark blue dots), RTTs versus Observer 2 (grey triangles), and for Observer 1 versus Observer 2 (light blue squares), respectively. Each dot, triangle, and square represents one fraction for one patient. RTTs = Radiation therapists. Obs = Observer.

**Fig. 4:**
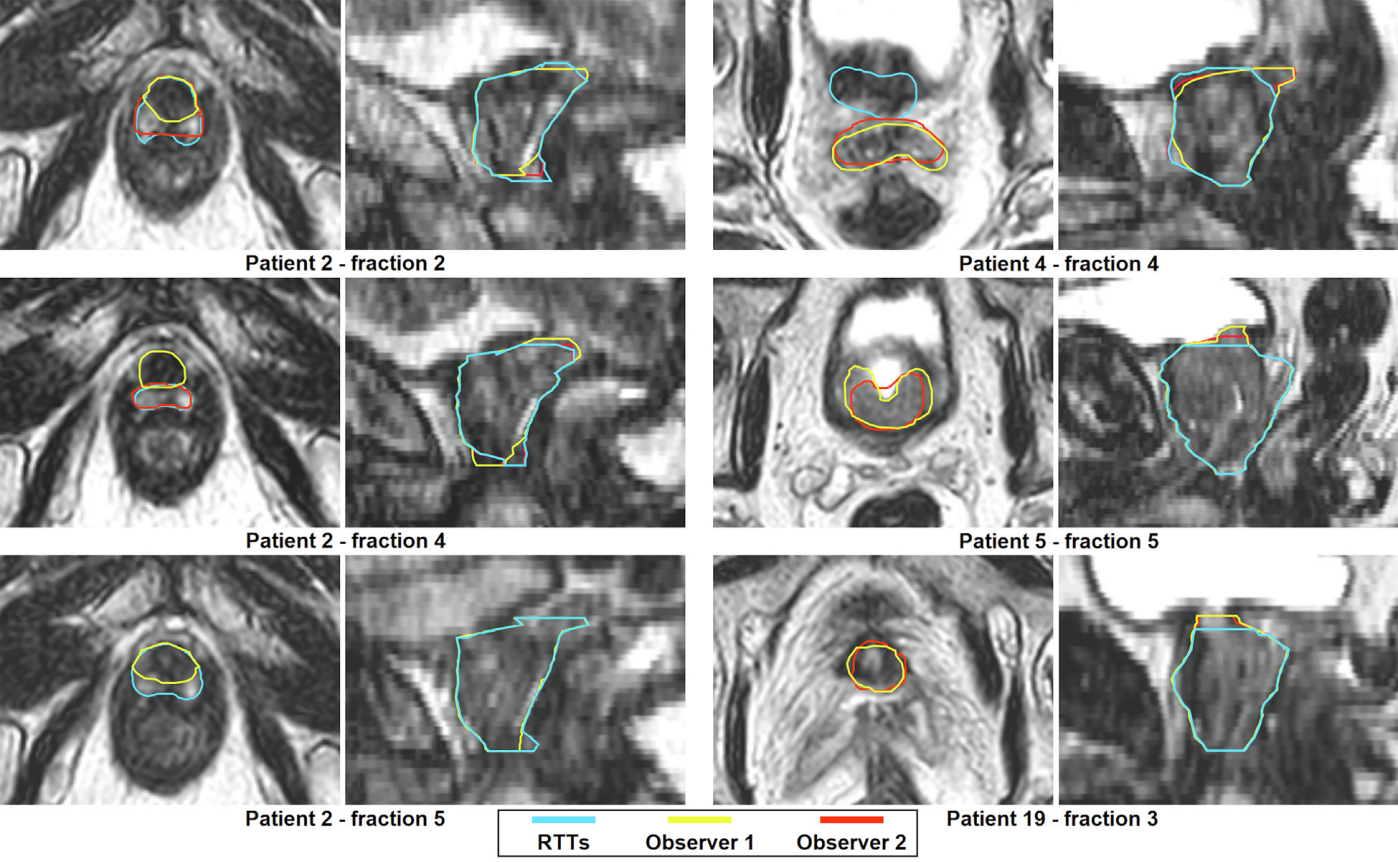
Examples of outlier cases. Transversal (left) and sagittal (right) images with CTV contours by RTTs (blue) for the seven fractions that were judged to need larger, potentially clinically relevant, adaptations, as judged by Observer 3, next to the adapted contours by Observer 1 (yellow) and/or Observer 2 (red). For Patient 19, fraction 3 and 5 are not displayed separately, since all contours were almost identical (shown here: fraction 3). For Patient 2, for fraction 2, 4, and 5 the CTV contour by the RTTs included the neurovascular bundle at the apex. For Patient 4, fraction 4, the seminal vesicles were missed in the CTV contour by the RTTs and the CTV contour was too wide towards the base and ventrally at the mid-prostate. For Patient 5 and 19, a larger part of the base of the prostate was left out. RTTs = Radiation therapists.

**Fig. 5:**
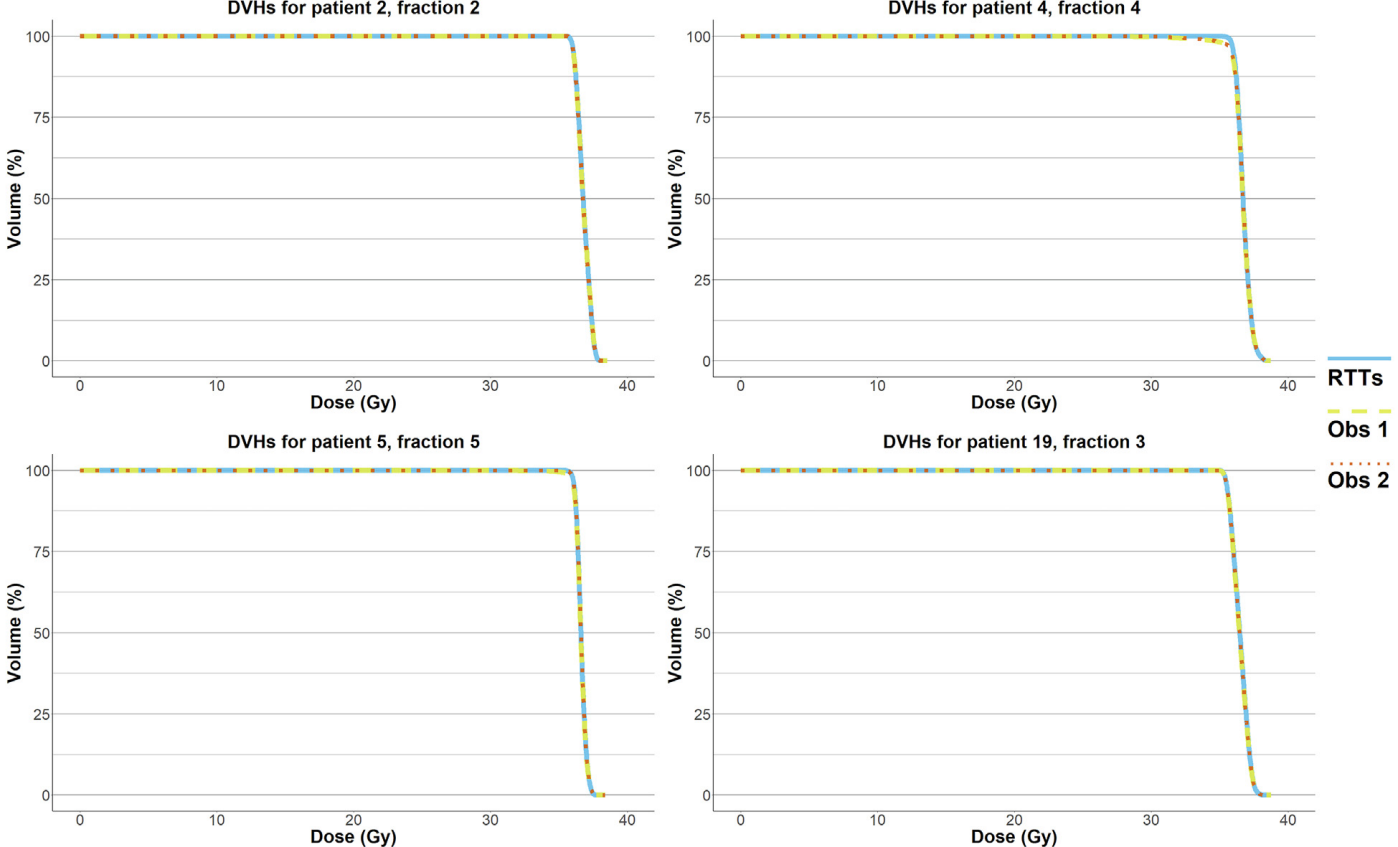
Dose-volume histograms. Dose-volume histograms (DVHs) for the CTV for four exemplary ‘outlier’ cases as judged by Observer 3. Dose distributions are based on the online (RTTs-contour based) dose plan as delivered to the patient. DVHs are given for the RTTs CTV contour (solid blue line), the adapted CTV contour by Observer 1 (dashed yellow line), and the adapted CTV contour by Observer 2 (dotted orange line). RTTs = Radiation therapists. Obs = Observer.

The contour adaptation times for the RTTs are presented in Supplementary Fig. S1. Mean (SD) contour adaptation time was 12.6 (±3.8) minutes. Adaptation results from the two independent observers are presented in Table 1. Observer 1 and 2 adapted 60 (50.0%) and 58 (48.3%) contours, respectively, while 50 (41.7%) contours were adapted by both. Observer 1 adapted none of the contours in seven patients (23.3%). For observer 2, this was the case in ten patients (33.3%). Most adaptations were performed in the apex and base region of the prostate and generally consisted of adjusting, adding, and/or removing one to three slices. Fig. 2 shows the relative volume differences per patient by observer, showing a median (interquartile range [IQR]) relative volume difference of 9.5% (4.3–13.6) in the RTTs group, 9.1% (4.4–12.7) for Observer 1, and 9.3% (4.5–13.0) for Observer 2. Median (IQR) interobserver DSC between RTTs and Observer 1, RTTs and Observer 2, and Observer 1 and 2 was 0.99 (0.98–1.00), 1.00 (0.98–1.00), and 1.00 (0.99–1.00), respectively (Fig. 3). RTTs contours from fraction two to five were acceptable for clinical use in 113 (94.2%) fractions as judged by Observer 3. Fig. 4 shows the seven remaining ‘outlier’ fractions in which larger adaptations were needed. DVHs for four exemplary outlier cases are displayed in Fig. 5 and CTV D99% is presented in Supplementary Table S1. Significant CTV under dosage was observed for one of the seven outliers (patient 4, fraction 4), with a D99% for the adapted CTV contours of 33.5 Gy (Observer 1) and 33.8 Gy (Observer 2) compared to 35.8 Gy for the RTTs contour.

**Table 1:** Total number of CTV contours in which adaptations were performed and number of adapted CTV contours per anatomical location, separately for independent Observer 1 and 2 (total number of contours/fractions = 120).

	Observer 1		Observer 2	
	Number of contours	Percentage of all fractions (n = 120)	Number of contours	Percentage of all fractions (n = 120)
Adaptations performed	60	50.0%	58	48.3%
Location of adaptation				
*Apex*	26	21.7%	37	30.8%
*Base*	39	32.5%	30	25.0%
*Mid-prostate*	5	3.3%	2	1.7%
*Seminal vesicles*	3	2.5%	4	3.3%

## 4 Discussion

In this study we evaluated the CTV contours, adapted and approved by RTTs, that were used for clinical treatment of PCa patients on an MR-Linac. The contours were evaluated using objective parameters and subjective clinical judgement. Interobserver DSC analysis showed high agreement and little to no impact of the adaptations performed. Contours were judged to be clinically acceptable without any adaptations in 94.2% of the fractions. DVHs analysis of the remaining cases showed that for only one case (one fraction), a significant impact on the CTV coverage occurred.

To our knowledge, no other published studies have evaluated the clinical feasibility and acceptability of online contour adaptation by RTTs on an MR-Linac. Several reports were published on first clinical experiences of PCa treatment using MR-Linac systems [11], [12], [13]. Most of these used a similar ATS workflow, with only Alongi et al. specifically reporting that physicians performed the manual contour adaptation [11]. Also, no other studies have evaluated contour adaptation variability in similar clinical circumstances. Pathmanathan et al. reported on interobserver variability in PCa contouring for MR-guided radiotherapy [14]. Five physicians delineated the prostate in ten patients on the same T2-weighted MR-image, showing a median DSC of 0.94 (IQR 0.93–0.95). These results were acquired with a different method, using one MR set per patient, and completely new delineations by physicians in an offline setting, thus making it difficult to compare with our results. Another study with similar methods to Pathmanathan et al. reported a slightly lower median DSC of 0.88 [15].

Adaptation times were generally longer and showed more variation in earlier patients compared to patients that were treated at a later date, with a mean (SD) adaptation time of 14.4 (±3.8) minutes in patient 1–10 and 9.5 (±1.4) minutes in patient 21–30 (Supplementary Fig. S1). This might partly be explained by a learning effect. There was no clear difference in the number of RTTs that were involved per patient in those with more and those with less variation (results not displayed). Our contour adaptation times are in line with Bertelsen et al., reporting a median adaptation time of 11.5 min (range 1–24 min) in their patients treated on an MR-Linac [12]. While contour adaptation times are generally comparable to those observed in physicians, this still raises a concern regarding anatomical changes that might occur during contour adaptation. This problem arises from the applied ATS workflow and the software used for warping the contours to the daily MR image. In the current workflow, a PV scan is obtained at the end of recontouring. In case of major anatomical differences, an ATP step can be applied, or it can be decided upon to redo the contours (ATS step) on the PV scan, i.e., in case of a large gas pocket in the rectum. Presently, we work towards a significant reduction of contour adaptation times by improving the accuracy of the propagated contours, potentially removing the need for contour adaptation altogether.

We found a median relative delineated volume difference of 9.5% between the first and subsequent fractions in the RTTs group and adaptations by Observer 1 and 2 did not have great impact on these volume differences. Most patients (22/30) showed only increased contour volumes in fraction two to five compared to the first fraction. However, we could not identify a trend in the prostate volume over the course of treatment. Firstly, it could be that there is a tendency to enlarge the propagated contour instead of ‘shaving off’ parts where the contour is too large, with the aim of not missing any part of the prostate. Secondly, there could be an actual increase in prostate volume over the course of radiotherapy treatment. Several reports have been published with varying results [16], [17], [18], [19]. While some studies showed an overall reduction of prostate volume at the end of treatment, these patients were treated with ≥ 38 fractions and a maximum fractional dose of 2.0 Gy [16], [17], [18]. Both King et al. and Nichol et al. reported an initial increase early in the course, the latter reporting an volume increase up to 34% [16], [17]. Gunnlaugsson et al. reported on volume changes in patients treated with extremely hypofractionated radiotherapy (7x6.1 Gy) [19]. They showed a mean increase of 14% mid-treatment and 9% at the end of treatment. These results could support our findings of increased volumes for all fractions compared to the baseline volume for the majority of patients. Still, we cannot conclude that the volume changes we observed are completely due to actual prostate volume changes, instead of (in part) interobserver variability.

Visual inspection of the contours showed some variation mainly in the apex and base region of the prostate and this was confirmed by the adaptations by Observer 1 and 2. While adjustments were made in about half of the fractions, these adjustments generally consisted of adjusting, adding, and/or removing one to three contour slices. Both the apex and base of the prostate are sometimes poorly visible on the T2-weighted MR scans that are currently used in our clinic for daily imaging and contour variability could be reduced with enhanced image quality. As stated before, these are the same areas that have been characterized in literature as being prone to interobserver variability [9], [10]. Although no statistical testing was performed, the recalculated DSC clearly reflect that the adjustments did not impact DSC in a significant way. Most interobserver DSC values were > 0.95, which is comparable to interobserver variability as discussed earlier [14]. For the fraction with lowest interobserver DSC (0.91 for patient 4, fraction 4, as visualized in Fig. 3), the seminal vesicles were partly missed in the CTV delineation. The high overall interobserver DSC can be explained by the relatively small (volume) changes that have been made. The question remains whether or not these adaptations have clinical consequences. In case of the current 5 mm PTV margin, one can argue that these adaptations mostly fall well within these margins. Thus, especially when taking the dose-gradient of external beam radiotherapy into account, these minor adaptations are not likely to influence the target coverage in a significant way. This view might change when smaller margins are being used. In seven fractions (5.8%), contour adaptations by Observer 1 and 2 were larger and potentially clinically relevant, as judged by Observer 3. To estimate the effect on the CTV dose for these seven fractions, we calculated DVHs for all CTV contours using the RTTs contour-based online dose distribution (Fig. 5). Also, we calculated D99% for the CTV (Supplementary Table S1). For just one fraction (patient 4, fraction 4), the adapted CTV contours showed clear under dosage near the seminal vesicles. We have to keep in mind that these numbers are all scaled to the complete five fraction scheme. Since this only occurred during one fraction, these effects would be smaller in reality.

There are several strengths to our study. Firstly, we have included delineation data from 30 patients, covering 150 fractions, created in the online MR-Linac setting with corresponding time pressure. Secondly, the retrospective nature of our study implicates that RTTs were not aware of the study. Hence, their efforts during contour adaptation were not influenced. Thirdly, contours were not only assessed by numerical parameters, but a blinded judgement of the clinical acceptability and need for adaptations of the contours was also part of our evaluation. Together with the DVHs analyses, this allows insight into potential clinical implications.

Conversely, a limitation of the study is the lack of a ground truth CTV contour for each of the fractions. We therefore chose to perform an interobserver comparison. Of course, both the adaptations by Observer 1 and 2, as well as the judgement by Observer 3, all reflect interobserver variability and we think that the clinical implications are limited. Only one of the seven fractions that were judged to need larger adaptations with potential clinical implications, showed a significant impact on the CTV coverage. Adding to this, we only calculated DSC and relative volume differences. Many other mathematical parameters exist that might help better understand how large the variation is between different observers, such as Hausdorff distance and mean absolute surface distance [14], [15]. However, these parameters, just like DSC, do not always correspond with clinical applicability or relevance, since a high DSC does not exclude the possibility of clinically relevant differences in a small part of the contour [20]. We therefore have chosen to focus on two numerical parameters and the blinded clinical judgement by three physicians, with the inherent limitation of subjectivity. Finally, we have only assessed potential dosimetric effects for the CTV coverage and did not assess any effects on OAR doses, as this was not the primary goal of this study.

## 5 Conclusions

Concluding, based on our evaluation of both DSC and clinical judgement, CTV contours that are adapted and approved in the online MR-Linac setting by RTTs are well-suited for radiotherapy treatment of PCa. Adaptations were mostly performed in areas that are known for their interobserver variability and clinical implications are thought to be minimal. The few outliers that were observed, were relatively small and mostly occurred only for a single fraction. The transition of this task from the treating radiation oncologist to the RTTs is feasible when RTTs are sufficiently trained and confident in their new task.

## Declaration of Competing Interest

The authors declare that they have no known competing financial interests or personal relationships that could have appeared to influence the work reported in this paper.
