# Supplementary material for: Evaluation of daily online contour adaptation by radiation therapists for prostate cancer treatment on an MRI-guided linear accelerator
Source: Clin Transl Radiat Oncol. 2021 Jan 14;27:50–6. doi: 10.1016/j.ctro.2021.01.002 (PMC7822780; doi:10.1016/j.ctro.2021.01.002)
Supplement: Supplementary data 1 [file mmc1.pdf]

Supplementary material

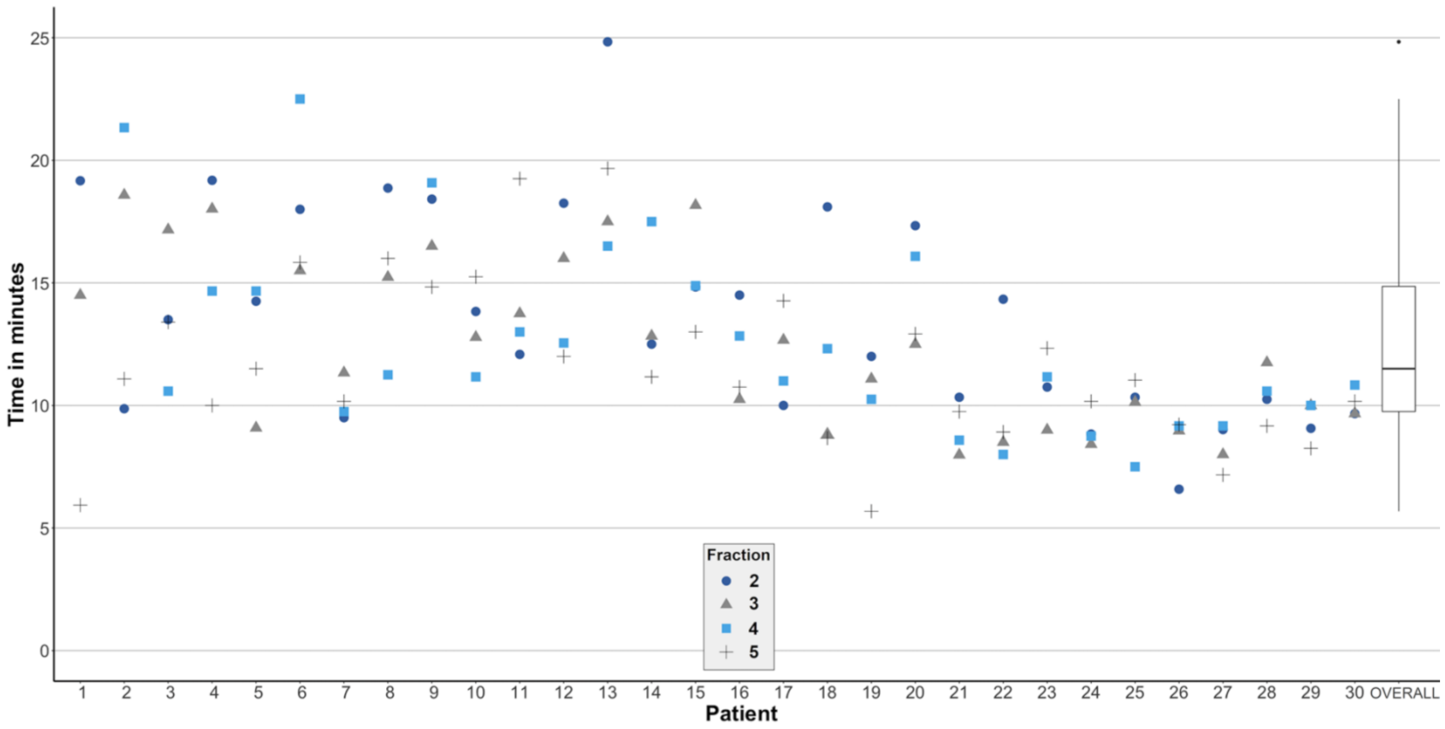

Figure S1 *Plots of contour adaptation times by the radiation therapists (RTTs) for fraction two to five for each patient separately and for all patients and fractions combined ("OVERALL"). For "OVERALL", the white boxplot box indicates the median (black horizontal bar), upper and lower quartile (vertical borders of boxes), and interquartile range (IQR). Black tails (error bars) indicate the lowest and highest value that is within the minimum and maximum value (minimum = lower quartile - 1.5*IQR and maximum = upper quartile + 1.5*IQR). Outliers are presented as black dots. For patient 1 the contour adaptation time was missing for fraction 4.*

Table S1 *Dose to 99% of the CTV (D99%) for the seven outlier fractions that needed potentially clinically relevant adaptations as judged by Observer 3, separately for the radiation therapists (RTTs), Observer 1, and Observer 2. For patient 2, fraction 5, no adaptations were performed by Observer 2.*

		**RTTs**	**Observer 1**	**Observer 2**
**Patient**	**Fraction**	**CTV D99% (Gy)**	**CTV D99% (Gy)**	**CTV D99% (Gy)**
2	*2*	35.86	35.85	35.85
2	*4*	35.80	35.78	35.80
2	*5*	35.64	35.64	*NA*
4	*4*	35.76	33.52	33.82
5	*5*	35.9	35.65	35.88
19	*3*	35.3	35.28	35.28
19	*5*	35.16	35.15	35.16
